# A multi-method study on GPs’ perspectives on Lipoprotein(a) as an actionable cardiovascular risk factor

**DOI:** 10.3389/fmed.2026.1859808

**Published:** 2026-06-12

**Authors:** Wann Jia Loh, Linh Thai, Yufan Yang, Jonathan Yeo, Bik Ling Poon, Andy Wong, Roy K. L. Teow, Elaine Lum

**Affiliations:** 1 Department of Endocrinology, Changi General Hospital, Singapore, Singapore; 2 Duke-NUS Medical School, Singapore, Singapore; 3 Yong Loo Lin Medical School, National University of Singapore, Singapore, Singapore; 4 Health Services Research & Population Health, Duke-NUS Medical School, National University of Singapore, Singapore, Singapore; 5 Tsinghua University School of Medicine, Beijing, China; 6 Department of Pharmacy, Changi General Hospital, Singapore, Singapore; 7 Singhealth DOT Primary Care Network, Singapore, Singapore

**Keywords:** family medicine, general practitioners, genetic lipid disorder, LILAC, Lipoprotein(a), Lp(a), primary care, qualitative study

## Abstract

**Introduction:**

Elevated Lipoprotein(a) [Lp(a)] is a common hypercholesterolaemia disorder that requires continuity of care from primary care doctors, yet elevated Lp(a) is a highly neglected condition globally. Here, we aimed to explore general practitioners’ (GPs’) perspectives and practices on the detection and management of elevated Lp(a) and to assess the impact of LILAC-for-Lp(a) training on GPs’ confidence in managing Lp(a).

**Methods:**

A total of 18 general practitioners (GPs) participated in the focus group discussions. Pre- and post-education questionnaires were administered to 50 GPs to assess changes in perceptions after education.

**Results:**

The median age of the focus group participants was 55 years, and 33% were women. Only two GPs were routinely testing for Lp(a) in patients at high cardiovascular risk. The analysis generated eight categories under four themes: (i) current context, (ii) patient selection for testing, (iii) barriers and enablers, and (iv) patient acceptance of testing. Although the GPs agreed that elevated Lp(a) levels should ideally be included in routine cardiovascular risk assessment, the critical barriers to integrating Lp(a) testing were the absence of nationally approved management pathways, out-of-pocket costs, low public awareness, and major knowledge gaps among healthcare providers. Key strategies include improving training and launching a national implementation programme with cost subsidies. Pre- and post-forum questionnaires collected from 50 GPs showed that the LILAC-for-Lp(a) educational framework had positively changed their perspectives on testing Lp(a) and improved their confidence in managing Lp(a) (*p* < 0.001).

**Conclusion:**

In conclusion, the barriers to testing and managing Lp(a) among GPs include an unclear management pathway and insufficient training. However, the LILAC-for- Lp(a) educational framework helped shift the GPs’ mindsets positively.

## 1 Introduction

Elevated Lipoprotein(a) [Lp(a)] is an established inherited hypercholesterolaemia condition that is recognized globally by scientific and medical bodies as an independent risk factor for atherosclerotic cardiovascular disease and calcific aortic valve disease (1). Multiple lipid and cardiovascular consensus statements, including those from the European, Canadian, and National Lipid Associations, recommend that Lp(a) testing not be limited to high-risk individuals and that all individuals be tested at least once in their lifetime to enable early cardiovascular prevention measures (2, 3). Supporting this movement, the recent Brussels international declaration paper, written by scientific and medical experts as well as patient advocates, called for universal integration of Lp(a) testing and management into routine clinical care (4). However, despite affecting about 20% of the global population, this condition remains severely underdiagnosed; fewer than 1% of individuals at high cardiovascular risk are tested, and many with elevated Lp(a) are not appropriately managed worldwide, including in Asian populations (5–9).

The views of working general practitioners (GPs) on Lp(a) are critical, as 80% of Singapore’s primary healthcare is provided by private-sector GPs, while 20% is managed in polyclinics (multi-doctor practices) under the public health sector (10). Beyond health screening and chronic disease management, general practice plays a crucial role in detecting and monitoring hypercholesterolaemia, as well as in conducting cascade testing for inherited lipid disorders such as familial hypercholesterolaemia and elevated Lp(a), both of which carry a 50% inheritance risk (11–13). However, there is a paucity of published literature on GPs’ perspectives on Lp(a).

Our work includes a recently published qualitative study of interviews with cardiology professionals, including cardiologists, cardiology-dedicated pharmacists, and nurses. We found that critical barriers to implementation of testing and management of Lp(a) in acute hospitals are the insufficient training in management of Lp(a) and misperceptions of Lp(a) as a non-actionable condition, leading to low confidence of healthcare professionals to test and manage Lp(a) (5). Additionally, enablers include user-friendly decision aids, adequate training and education, and the integration of Lp(a) into clinical pathways (e.g., acute myocardial infarction pathways) (5). Thus, a novel concept, #LILAC-for-Lp(a), was created and launched to aid clinical practice; LILAC is an educational method, a cognitive aid for healthcare professionals, and a tool in this implementation science work (11). The five salient points in LILAC encompass the principles for managing elevated Lp(a). This includes stratification of an individual’s overall cardiovascular risk with the inclusion of Lipoprotein(a) and other dyslipidaemias (1st letter L), empowerment for lifestyle changes (letter I), management principles (the letters L and A), and cascade testing (letter C) (14–16).

In this study, we aimed to explore GPs’ perspectives and practices on the detection and management of inherited hypercholesterolaemia, particularly elevated Lp(a), using a qualitative focus group study. We also investigated whether their perspectives on Lp(a) changed after learning about the LILAC-for-Lp(a) educational framework through short educational videos.

## 2 Methods

### 2.1 Study setting and recruitment

The focus groups were conducted at the Medical Centre of Changi General Hospital as part of the GP Lp(a) Forum on the afternoon of 7 December 2024. This free-to-attend education forum was designed as a continuing medical education (CME) event for GPs on Lp(a) with limited seating for 30–35 participants, with the option to participate in a focus group discussion. The event was advertised via posters and e-posters to more than 2,000 GPs registered in private practices across Singapore. Eligible participants for the focus group discussion were GPs who were actively practising, were below 75 years of age, and had a minimum of 1 year of working experience as a GP. The intended total sample size for the focus groups was 20–25 participants.

The forum was structured as follows: Two focus group discussions were held with consenting eligible GPs, each lasting 1 h (*n* = 18). The GPs who participated in the focus group discussions were given SGD$100 as remuneration for their time. After lunch, a series of talks by specialists was held. The first talk was “Clinical significance of Lp(a) in the world and in Singapore” (WJL, an endocrinologist and head of the Lipid Unit), followed by “Lp(a) and cardiovascular disease” (CY, cardiologist). Subsequently, two LILAC-for-Lp(a) short educational videos (9 min each) created by WJL were shown. Pre- and post-video questionnaires were administered to 20 GPs. At the end of the forum, all attendees’ verbal and written feedback was collated. All 26 GPs, including retiring and late-arriving GPs who attended the CME event, earned CME points. Another 30 GPs who did not attend the educational forum watched educational videos and completed pre- and post-questionnaires, bringing the total number of respondents to 50. The local ethics committee, SingHealth Centralised Institutional Review Board [CIRB], approved this study under approval numbers CIRB 2023-2,429 and CIRB 2025-0274.

### 2.2 Focus group discussions

The questions used in the focus groups were based on the Theoretical Domains Framework adapted from our previous qualitative study of pre-implementation barriers to Lp(a) testing and management in hospital patients at high cardiovascular risk (5). For these GP focus groups, the discussion began by eliciting their views on the diagnosis and management of various hereditary lipid disorders and hypercholesterolaemia. Additional questions sought their opinions on who in primary care should manage elevated Lp(a), referral threshold, barriers to care, cascade testing, and whether Lp(a) should be routinely tested.

The 18 GPs who consented to participate in the focus group were randomly assigned to two groups of similar size. Focus group 1 (FG1) and focus group 2 (FG2) were led by an external facilitator. Observers for FG1 were BLP (pharmacist), YY (medical student), and CY (cardiologist). Observers for FG2 were JY (pharmacist) and AW (endocrinologist). To reduce potential response bias, WJL, who is a frequently invited speaker on lipid disorders, was not an observer during the focus group discussions. The two focus groups were held concurrently in separate meeting rooms at the same venue, each lasting approximately 1 h. Focus group discussions were audio-recorded, de-identified, and transcribed verbatim with Jeffersonian annotations by BLP, YY, and JY. Additionally, one or two paragraphs of general impressions were provided by the following observers: BLP, YY, and JY.

### 2.3 Questionnaires

A short questionnaire, similar to that previously described in our published work, was administered before and immediately after LILAC-for-Lp(a) education to assess self-rated confidence and perceptions of Lp(a). GPs responded via a Quick Response (QR) code to Microsoft Forms (*n* = 20) (14). Additional questions, not included in our prior work, assessed GPs’ self-rated confidence in taking history and counselling patients about elevated Lp(a) among those attending the in-person educational forum and viewing the LILAC educational videos (*n* = 20). Unlike our prior work, which included only a single, succinct 9-min educational video for 500 participants working in a single tertiary hospital, this current educational forum for GPs included an additional short educational video based on case scenarios involving elevated Lp(a) (17, 18). Via an open invitation to GPs, an abbreviated version of the questionnaire was given to 30 additional GPs before and after they watched the 2 educational videos via a QR code.

### 2.4 Data analysis

#### 2.4.1 Qualitative analysis of focus group discussions

The researchers (EL, LT) involved in the data analysis reflexively contextualized the coding decisions and questioned the assumptions given their multi-disciplinary backgrounds, which were implementation science (EL), clinical pharmacy (EL), health services research (EL, LT), qualitative research (EL, LT), life sciences (LT), and psychology (LT). A constructivist approach was used to understand the subjective meanings and social constructions of GPs’ thoughts on lipid management and Lp(a) testing in a primary care setting. The data were analysed following Gale et al.’s (19) approach. EL and LT independently read the transcripts and observers’ general impressions (one to two paragraphs provided by BLP, YY, and JY) to familiarize themselves with the dataset. The paragraphs provided by the observers were used to examine the alignment (or otherwise) of our interpretation regarding the general direction of discussion.

LT conducted inductive coding using Microsoft Word® (Microsoft 365, version 2,510) with iterative and regular discussions with EL. Disagreements in coding were resolved through discussion and re-reading of transcripts to reach a consensus. A codebook was constructed and refined during the analysis. The final codebook is enclosed as a Supplementary material. The codes were clustered into higher-order subcategories, categories, and themes. Although thematic saturation could not be assured, as only two focus groups were conducted, the data from these two discussions were sufficiently coherent and in-depth to address the research questions.

#### 2.4.2 Quantitative analysis of questionnaires

A paired *t*-test was used to analyse the mean change in the Likert scale scores, comparing pre- and post-scores (maximum score of each is 10 points) after LILAC education. The visual analogue scores a total of 50 GPs completed both pre- and post-education questionnaires. Pearson’s correlation was used to measure the relationship between the years of experience of the GP and each question on self-rating of confidence. A *p*-value of <0.05 was considered statistically significant.

## 3 Results

**Figure 1:**
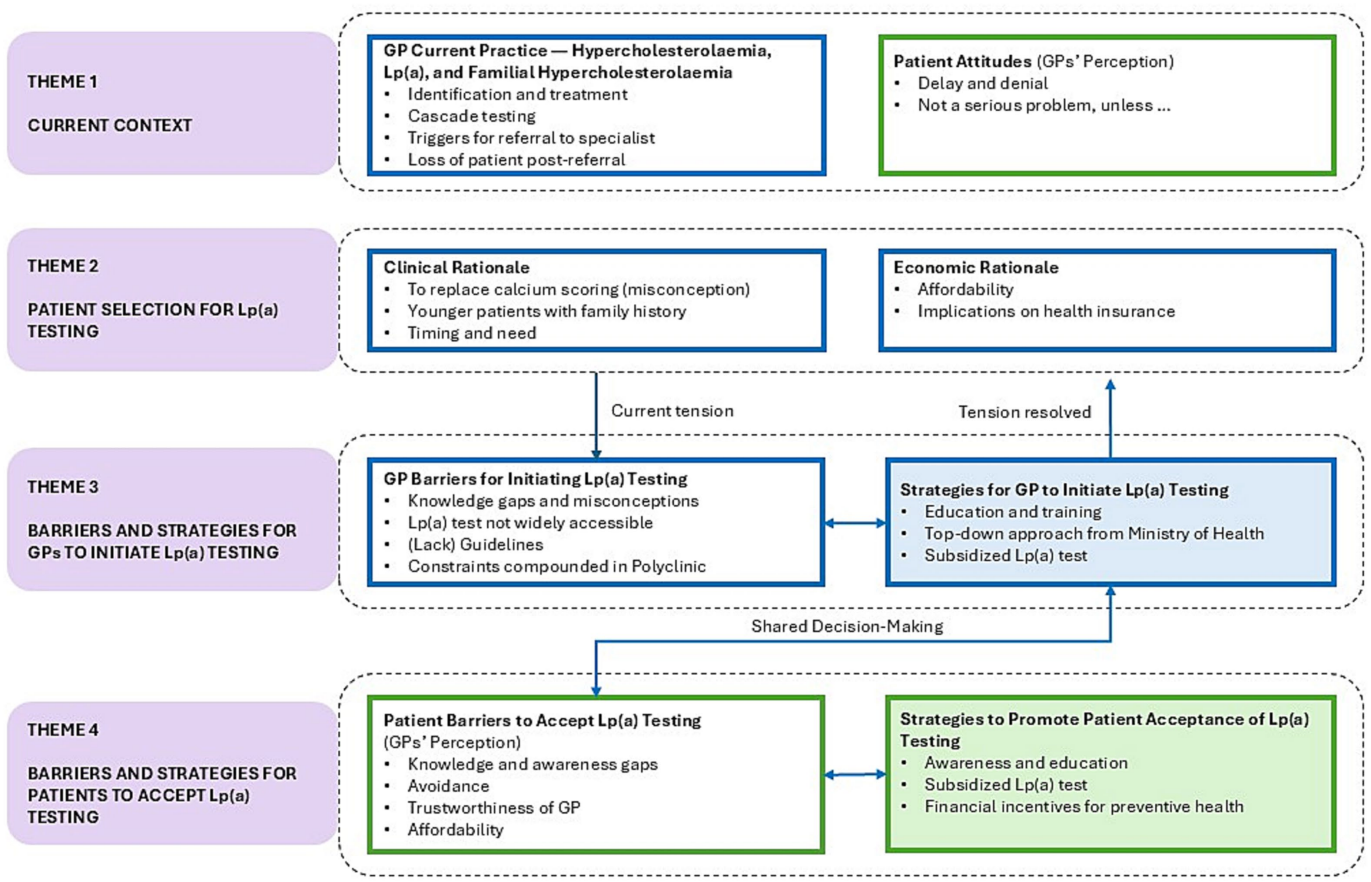
The four themes, categories, and subcategories were ascertained from focus group discussions with GPs on the testing and management of Lp(a).

A total of 18 GPs consented to participate in focus group discussions. The ages of the GPs ranged from 39 to 69 years (median, 55 years); 33% (6/18) were women; and clinical experience as GPs ranged from 3 to 38 years (median, 30 years; missing data, *n* = 5). Qualitative data analysis generated four themes and eight categories. The themes are: (i) Current context; (ii) Patient selection for Lp(a) testing; (iii) Barriers and strategies for GPs to initiate Lp(a) testing; and (iv) Barriers and strategies for patients to accept Lp(a) testing. The findings are schematically summarized in Figure 1.

### 3.1 Theme 1: current context

This theme captures current practices among GPs in managing common hypercholesterolaemia, familial hypercholesterolaemia, and elevated Lp(a), as well as their observations of patient attitudes towards these conditions.

#### 3.1.1 GP current practice

##### 3.1.1.1 Identification and treatment of hypercholesterolaemia disorders, including familial hypercholesterolaemia

GPs reported two common scenarios for identifying hypercholesterolaemia in clinical practice, namely, during health screening or when ordering additional tests for patients with known chronic conditions such as diabetes and hypertension.

“*[…] we see a lot of cases that come in [for] health screening […], and we pick them up incidentally. Although [if] they already have concomitant hypertension and diabetes […] we always do [lipid tests] as a routine test for them*.” (FG1 P9)

Treatment of hypercholesterolaemia is based on either Singapore or international clinical guidelines. *“[…] we base our treatment on guidelines […] recognized in Singapore and internationally. So, it’s easy [to decide on treatment] because we follow the guidelines*.” (FG1 P9).

GPs indicated that they would be aggressive in treating hypercholesterolaemia, regardless of whether the patient has familial hypercholesterolaemia. This is especially the case if the patient is young.

“*The academic side, there will be different kinds of hereditary familial hypercholesterolaemia, but on our GP side, as far as I’m concerned, I’m just trying to bring it [LDL] down […] I will treat aggressively […] my aim is […] to bring down the LDL […] whether it is familial or not […] is academic. […] young people with cardiovascular diseases, then the urgency is there.*” (FG2 S2)

A patient’s family and medical history would be considered when determining the degree of aggressiveness required for lipid-lowering therapy.

“*[after assessing family history] so you already can tell, this is probably familial, and they probably will not respond to lifestyle modification alone. Another set of people that I find with high LDL is hepatitis B carriers. Their livers are damaged […] so their lipids are high […] go straight [treat with] into statin.*” (FG2 S8)

The GPs were asked about their approach to managing a hypothetical patient with elevated Lp(a) levels after hospital discharge. GPs stated that they would follow the specialist’s management plan, with the expectation that the patient would have an annual check-in with the specialist.

“*Usually the specialist will start treatment, like, maybe increase the statin […]. Then you follow up with the recommendations from the specialist. Carry on the medicine and then you monitor the LDL; and of course, you have to be aware that the LDL is supposed to be lower than normal, because you have an Lp(a) positive [patient]. […] I’m sure they also have a yearly check-up with their cardiologists.*” (FG1 P9)

##### 3.1.1.2 Cascade testing

GPs typically refer patients with familial hypercholesterolaemia to the hospital for cascade testing. Perhaps due to the relative novelty of cascade testing for family members of an index patient with elevated Lp(a), participants’ discussions centred on the acceptability of cascade testing.

“*I think it depends on individual families, how they value their health, you know. Do they see this as important to them or not. […] I mean, you can tell the patient to invite the family members, but of course, you also must tell them the risk of the insurance […] if positive [elevated Lp(a)]. […] let them decide; whether […] health is more important or insurance premium is more important.*” (FG1 P9)

The willingness of GPs to recommend cascade testing is also determined by the likelihood of detecting elevated Lp(a) levels in family members. “*I want to know […] what is the chance that the patient’s brother or sister will have a positive [elevated] Lp(a), also, and the second-degree relative. […] if you tell me that even among siblings it’s only 30%, then I would be a bit hesitant to ask […] family members to come for screening.*” (FG1 P2).

##### 3.1.1.3 Triggers for referral to a specialist

GPs would refer patients to specialists or lipid clinics if hypercholesterolaemia remained uncontrolled or if multiple comorbidities and other modifiable risk factors were present.

“*If you can’t [manage/control patient’s hypercholesterolaemia], it’s still high, high, high; then you better let go [refer to specialist]*” (FG2 S1).

##### 3.1.1.4 Loss of patient post-referral to specialist

Unfortunately, the referral loop seemed to be “one-way.” The GPs were dismayed that they would lose the patient after referral to a specialist, even though they preferred to retain the patient.

“*Once we refer, they stay with the specialist. They don’t come back.*” (FG2 S3).

Specialists were perceived as referring patients to polyclinics rather than GPs. “*Normally, the specialists do not refer back to us. […] when they discharge the patient go to polyclinic, they just skip the GP when we are the first one to refer to them.*” (FG2 S3).

However, the failure to refer patients back to their GPs may be due to patient choice: “*[…] specialist will give the patient a choice of going back to the family doctor or the polyclinic.*” (FG2 S2).

#### 3.1.2 Patient attitudes towards hypercholesterolaemia

##### 3.1.2.1 Delay and denial

GPs reported that some patients with hypercholesterolaemia, especially younger patients, were reluctant to take cholesterol-lowering medications without attempting lifestyle changes first (this aligns with lipid management guidelines for patients at low risk of cardiovascular events). There was also an element of denial in some patients.

“*[…] yes, the lipid levels are high. Then they just say, ‘Oh, give me three months,’ and they try to […] do some lifestyle changes, you know? But more often than not, we don’t see them coming back. […] they don’t want to put a stamp at such a young age that they have high cholesterol levels.*” (FG2 S6)

Patients may also be reluctant to start lipid-lowering medications owing to potential side effects and concerns about the lifelong need for these medications. Patients may also be unwilling to continue lipid-lowering medications once their cholesterol levels are reduced.

##### 3.1.2.2 “Not a serious problem, unless …”

Due to the high prevalence of hypercholesterolaemia in Singapore, some patients do not consider high cholesterol to be a serious problem. “*They don’t think […] having [high] cholesterol in the family is like having cancer. […] they would not act on it [high cholesterol] and be serious about it.*” (FG2 S3).

In contrast, patients with parents or close family members with relatively recent cardiovascular events, especially if hospitalized are more likely to be proactive in seeking cholesterol tests and management. “*Patients who have family members [with] cardio events […] they are a bit scared, and they would go for the screening.” (FG2 S7); and “if they have seen their parents in CCU [coronary care unit], then they will really go for that [screening], you know.*” (FG2 S7).

### 3.2 Theme 2: patient selection for Lp(a) testing

GPs were selective regarding which patients they would recommend an Lp(a) test to. Patient selection for Lp(a) testing is driven by two factors: clinical and economic rationales.

#### 3.2.1 Clinical rationale that was considered by GPs, including testing in patients with a personal or family history of cardiovascular disease

However, there was also a misconception that Lp(a) may be a helpful replacement for other modalities of cardiovascular stratification.

“*I don’t do [Lp(a)] for everybody. I do for those who are […] borderline high cholesterol […] whether you want to do calcium scoring to identify whether they are very high risk. But to do calcium scoring means we have to refer […] costs hundreds of dollars. So, I use Lp(a) [test] as a so-called replacement.*” (FG1 P7)

“*[…] family history wise, some very young person with heart events, then we will encourage the whole family to go for screening. […] if it is themselves having a heart attack below 50 [years old], then of course also ask him to ask family members to screen.*” (FG2 S3)

GPs would also assess the timing and necessity of an Lp(a) test on a case-by-case basis. For example, to individualize the timing of offering the Lp(a) test by addressing the patient’s known risky behaviors first:

“*[…] let’s say the guy has […] terrible cholesterol […] then you don’t need to do the Lp(a) first. […] this would be an unnecessary supplementary test. He’s already smoking 20 sticks and all that, so […] it’s not gonna be much of a difference.*” (FG1 P1)

And perhaps not to offer the Lp(a) test at all for patients known to be at high risk for cardiovascular events:

“*[…] if you already have the whole Christmas tree full of other risk factors already […] and his dad died at 30; then there’s no need to do Lp(a) [test]. You know that he’s very high risk already.*” (FG1 P1)

GPs also pondered whether the Lp(a) test may be unwarranted if treatment goals were already met: “*[…] how low can you go?*” (FG1 P7).

For patients who have not yet achieved their treatment goals, Lp(a) testing (if elevated) would provide further rationale for intensifying treatment, thereby reducing clinical inertia. This would also motivate reluctant or undecided patients to start lipid-lowering treatment.

#### 3.2.2 The economic rationale was two-fold, being the affordability of testing for patients and implications on insurance (“insurability” and insurance premiums)

##### 3.2.2.1 Affordability

GPs noted that some patients are either unwilling or unable to afford out-of-pocket costs for an Lp(a) test (currently costing between SGD 50 and 60, roughly equivalent to USD 40 and 50), especially those who rely on government subsidies for healthcare via the Community Health Assist Scheme (CHAS).

“*[…] my patients do not want to pay the extra money […]. So, they [patients] tell me […] whatever can be deducted, you know, from CHAS. By the time you do a blood test, […] the medication, already that is the [CHAS] limit. You hit the [CHAS] limit. I can’t really do [Lp(a) test].*” (FG1 P3)

“*[…] we are not doing it [Lp(a) test]. It’s not indicated at all. […] it’s not covered by Screen4Life [national health screening program rebranded to HealthierSG]*.” (FG2 S5)

Likewise, participants who practice in polyclinics do not order Lp(a) tests, as these are not subsidized currently: “*We do not do this kind of screening tests [Lp(a)] unless it’s from upstairs (Ministry of Health), because [then] it will be subsidized.*” (FG1 P7).

A GP (FG1 P3) encountered similar obstacles in providing care to patients as a panel clinic contracted by employers: “*Company [panel clinic], [patient] wasn’t very keen […] because it’s not part of our official package [of tests]. So, [patient] refuses to top up extra [for the Lp(a) test]. So, it’s not easy at this moment.*” Other GPs concurred and indicated that “because it’s not free” (FG1 P1 and P3), the majority of the patients are reluctant to be tested for Lp(a).

Patients who are (better educated and) from higher socioeconomic classes might be more amenable to Lp(a) testing and paying for the test. (Note: The participant is conflating “very educated” with higher socio-economic status and disposable income.)

“*But the very educated ones would choose to do my $400–$500 health screen, and they ask me […] ‘anything else you think I should do [test]?’ […] Let’s do an Lp(a). […] anyone who wants to buy my $400–$500 health screen, this additional $55 is okay.*” (FG1 P7)

##### 3.2.2.2 Implications on health insurance

Implications of Lp(a) testing on “insurability” and insurance premiums were discussed in relation to the index patient. This implication partially explains GPs’ reluctance to offer the test, as they anticipate their patients’ hesitancy to disclose it to family members or pay higher health insurance premiums that may occur, although this remains an unverified concern.

“*Because you may just subject [sic] your patient’s children from buying insurance in future. Because if they are all found [to have elevated Lp(a)], then the declaration problem. So, do you really want to do the test for them?*” (FG1 P8)

“*So, implications, insurance. […] When we write insurance report, […] ‘what are the abnormal tests that you have over the past year?’*” (FG1 P7)

“*So, the [elevated] Lp(a), […] they will insure you at a higher premium.*” (FG1 P1)

Despite the barriers discussed (see Theme 3), participants unanimously agreed that Lp(a) should be included as a routine component of cardiovascular risk assessment. However, participants robustly debated whether to delay offering patients Lp(a) testing until effective treatments to reduce Lp(a) became available in the market. The group concluded that such a delay was unwarranted, citing a historical example of cholesterol testing before effective medications were available.

### 3.3 Theme 3: barriers and strategies for GPs to initiate Lp(a) testing

This theme makes explicit the causes of hesitancy that prevent GPs from initiating Lp(a) testing, as well as strategies that would mitigate these barriers.

#### 3.3.1 GP barriers for initiating Lp(a) testing

##### 3.3.1.1 Knowledge gap

Lp(a) testing is new to GPs, and many were unaware of it or became aware of it only recently. Participants were largely not ordering Lp(a) tests currently, apart from one or two tests in each focus group. Those who were persuaded of the utility of Lp(a) for cardiovascular risk stratification began to order Lp(a) tests.

“*So, this is something new for us doctors who graduated maybe 20, 30 years ago. We’ve never even heard of Lp(a).*” (FG1 P9)

“*Super blind spot, super blind spot.*” (FG2 S7)

“*I heard two talks from cardiologists, so I’m pretty convinced about the [Lp(a)] utility.*” (FG2 S8)

“*I only learned about it during the recent CME at [hospital redacted], […] saying that everybody, once in a lifetime should do a Lp(a), to see where you stand and that got me interested. Lp(a) can be ordered through the Innoquest lab. […] So I start doing Lp(a) probably about a month ago*?” (FG1 P7)

Another knowledge gap is the interpretation of the Lp(a) test results. “*[…] so even if you give me a number, I do not know how to interpret it. So, I don’t do it [Lp(a) test].” (FG2 S3); and “[…] but we don’t know how to interpret, we do not know how to use it.*” (FG2 S3).

##### 3.3.1.2 Lp(a) test not easily accessible in primary care

GPs are currently not managing patients with elevated Lp(a). One of the reasons for this is that Lp(a) tests are not easily accessible: “*No, because the test is difficult to get […] it’s not readily available previously.*” (FG1 P5).

##### 3.3.1.3 (Lack of) Guidelines

GPs were reluctant to advocate Lp(a) testing because of the lack of officially sanctioned clinical guidelines from the Ministry of Health, Singapore (MOH).

“*[…] the Ministry hasn’t formalized [guidelines]. […] You don’t have anything [treatment pathway], […], you frighten the patient and what you going to do? So, we [GPs] can’t do anything about it.*” (FG1 P4)

“*As long as Lp(a) not in the main MOH ACE guideline [I won’t test/advocate]—it’s not a bottom-up grassroots movement. It has to be very top-down. You’ve got to convince the powers that be and not grassroots fodder like us.*” (FG2 S2)

“*[…] the money is from them [MOH], the KPI is from them; so, you ask us [GPs] also no point.*” (FG2 S1)

GPs in focus group 2 perceived that the government’s public health agenda currently focuses on diabetes rather than hypercholesterolaemia. GPs in focus group 1 perceived that the Ministry of Health would act quickly if convincing evidence was available for Lp(a) testing:

“*Depends on the significance of the risk, right? If the risk is very, very low, then everybody will just dilly dally right, there’s no real urgency, right? But if one day a study comes out that shows you that the risk is very, very high, then we [GPs] don’t have to do anything [don’t have to advocate]. […] it will automatically get done [directives from Ministry of Health], right? There will be mass info to the public.*” (FG1 P2)

The current lack of MOH clinical guidelines for Lp(a) testing and subsequent clinical management introduces uncertainties for GPs and raises anxieties about governance and medicolegal liabilities. GPs cited examples from the Healthier SG (Singapore’s national preventative health program) when discussing their anxiety about acting without clear clinical guidelines, including the fear of being found to be non-compliant should they be audited.

“*[…] and now with HealthierSG, they [MOH] are so sticky about step one to two and three. It is like following a real textbook […]*” (FG1 P7)

“*If you don’t follow, they will penalize you.*” (FG2 S5)

“*I think if we do that [Lp(a) test] out of the ordinary [currently not in HealthierSG], […] if they [MOH] do auditing, would they say something about this?*” (FG2 S5)

The fear of the potential legal repercussions of GPs acting without sanctioned guidelines is a stumbling block.

“*But if they [MOH] never say anything [no clinical guidelines] and you do it [Lp(a) test]; then the patient say ‘oh hey, […] take statin may cause diabetes.*’” (FG1 P4)

“*[…] if you do the test for them, even you give them free, the Lp(a), and later found to be positive, they can sue you in future. So, be very careful doing it because it’s close, akin to genetic testing.*” (FG1 P8)

In the current vacuum of the official Ministry of Health guidelines on Lp(a) testing and management, GPs could arguably use international guidelines. However, there is dissonance between international guidelines, and selecting which one to use is not straightforward.

“*Honestly, I don’t know. […] the Stanford people said that in America they don’t care [about Lp(a)], Europe they care a bit more. […] what can a GP do?*” (FG2 S1)

Regarding doctor−patient shared decision-making for Lp(a) testing, GPs perceived little evidence of this in practice. However, GPs emphasized the importance of respecting patients’ individual choices: “*[…] the other thing that we need also to consider is the individual choice, because if you try to say it’s important to do it, but people may be afraid, because there are people who tell me, “I don’t want to do it.”* (FG1 P3).

To mitigate these critical barriers, strategies that enable GPs to initiate Lp(a) testing with confidence are required. Participants spoke directly about the need for education and training, as well as guidelines from the Ministry of Health. These official guidelines would, in turn, confer legitimacy on billing Medisave or access to subsidies for Lp(a) testing.

#### 3.3.2 Strategies for GPs to initiate Lp(a) testing

##### 3.3.2.1 Education and training

Address the knowledge gap about Lp(a): “*some education […] for the GPs, the older GPs especially, who are not aware.*” (FG1 P9). GPs in the focus groups indicated that they would like more information and published evidence on the following: “*What is Lp(a)? How does it help in cardiovascular risk stratification? How would knowing that a patient has elevated Lp(a) levels change subsequent clinical management? What strategies and treatments are available now to address the increased cardiovascular risk of patients with elevated Lp(a)? Would the benefit of more aggressive treatment with statins outweigh the increased risk of developing diabetes?*”

Educational strategies targeted at GPs should aim to dispel the perception that Lp(a) testing confers only a theoretical advantage and that nothing can be done to reduce the risk of elevated Lp(a). An example of this misperception is as follows.

“*Because, you know, you can’t do anything about it; the risk is there [elevated Lp(a)] but exercise won’t change anything unless you have medication. But you don’t have medication.*” (FG1 P4)

Educational strategies for GPs should also include tools that they can use with their patients. For example, a “conversation script” or a consistent approach for how to discuss and arrive at a shared decision regarding Lp(a) testing.

“*[…] just once in your life [Lp(a) test]. That’s it. We will not repeat ever again, we’d know your risk factor already. […] So, maybe that will change their perspective about the money.*” (FG1 P9)

##### 3.3.2.2 Guidelines from the Ministry of Health

These guidelines should also be suitable for Singaporean populations.

“*[…] we need more Singapore experience on this, because these criteria are all international. […] quite a few patients I wanted to give—turns out MOH tells me not to give statin. […] So, I think we need something of [sic] authority […] MOH to come out with better criteria, because these may be all international criteria.*” (FG1 P8)

In addition, GPs would also like Ministry of Health guidelines on cascade testing for Lp(a) so they can “just follow, like the breast cancer guidelines, you know.” (FG1 P4).

##### 3.3.2.3 Subsidized Lp(a) test

Focus group discussions indicated that GPs perceived patients as reluctant to accept Lp(a) testing because of the out-of-pocket expenses they were unwilling to pay (see Theme 2, patient selection for Lp(a) testing).

The availability of the Ministry of Health guidelines would not only signal the legitimacy of Lp(a) testing to the general public and patients but also enable GPs to bill Medisave or CHAS (see Theme 2, patient selection for Lp(a) testing). This guideline-enabled billing mechanism would eliminate out-of-pocket expenses for patients by subsidizing the Lp(a) test.

### 3.4 Theme 4: barriers and strategies for patients to accept Lp(a) testing

This theme identifies barriers to patient acceptance of Lp(a) testing and strategies to mitigate them.

#### 3.4.1 Patient barriers for accepting Lp(a) testing

##### 3.4.1.1 Knowledge gap

The lack of awareness of Lp(a) among the general public and patients contributes to their reluctance to incur out-of-pocket expenses.

“*I think there’s an awareness issue, because the public may not know the importance of Lp(a), and they think it is just extra cost, you know. After all, you’ve already done the fasting lipid.*” (FG1 P9)

Creating awareness and educating the general public and patients regarding the significance of Lp(a) would help persuade them to pay for the test.

“*So, unless we can convince a patient that this [Lp(a)] is different […] and change their perspective […]. It’s not that they don’t want to pay [extra], they don’t know what this is for […]*” (FG1 P9)

##### 3.4.1.2 Avoidance

Avoidance has emerged as another key barrier. Even if patients understand the importance of the Lp(a) test, they might still resist it and prefer not to know the following:

“*[…] we also need to consider individual choice […] people may be afraid […] there are people who tell me ‘I really don’t want to know.*’” (FG1 P3)

In part, this resistance to Lp(a) testing might be explained by the desire to avoid emotional distress for themselves (and their families), as there is a genetic implication, unlike other blood tests:

“*[…] someone [patient] actually said, ‘after you do the testing for me, if it is abnormal, I have to start worry[ing] not just myself, [but for] my family, my children, and you know, everybody [then] needs to get tested.’ […] there are implications because it’s a genetic thing as well.*” (FG1 P3).

##### 3.4.1.3 Trustworthiness of GPs

Another barrier is trustworthiness. Whereas patients tend not to question tests ordered in hospital settings, this may not be the case with GPs. Patients implicitly trust public hospitals, which bolsters GPs’ arguments for Lp(a) testing in hospitals rather than in primary care settings.

“*[…] they trust the hospital, because after all it’s under the government.*” (FG1 P6)

“*[…] my patients go to the hospital ward, they get a lot [of] testing done, you know. [They] don’t say anything, because the hospital doctors make decisions for you, the specialist makes decisions for you.*” (FG1 P3)

Hence, GPs perceive that it would be relatively easy for hospitals to order an Lp(a) test for the patient: “*I think if it’s in the hospital setting, […] it’s easy to do an Lp(a). No problem. You just order it, part of the package. People don’t even know what is ordered for them.*” (FG1 P3).

In contrast, if GPs were to order an Lp(a) test, given the additional cost, they would need to justify to the patient why the test was required, thereby requiring additional time and effort. Time constraints are more acute in polyclinic settings.

“*Whereas for us, it would take a lot of time to explain, it takes a lot of effort to explain; and it’s not so easy.*” (FG1 P3)

The GPs observed that patients may be concerned about profit-driven healthcare. Hence, unlike not-for-profit public hospitals and polyclinics, where “*[…] they know that the money do not [sic] go to the polyclinic doctors for cares [sic]*” (FG1 P7), GPs needed to assure patients that the recommendation to do an Lp(a) test is credible and not simply profit-making.

“*So, sometimes I just turn to the page [Family Medicine newsletter]; I’ll underline and I’ll show them [the data/evidence]. And then, they recognize that because it’s from a recognized institution, they are a little bit more open to it [Lp(a) test].*” (FG1 P6)

##### 3.4.1.4 Affordability

The current cost of Lp(a) tests may be prohibitive for some patients. A reduction in the cost of Lp(a) testing would encourage its acceptance by both the general public and patients. “*[…] it may not be $55, maybe it’s only $25, you know. Then, it’s more acceptable to the masses.*” (FG1 P9).

#### 3.4.2 Strategies to promote patient acceptance of Lp(a) testing

The following strategies promote acceptance of Lp(a) testing among patients and mitigate the identified barriers.

##### 3.4.2.1 Awareness and education

Addressing the knowledge gap regarding Lp(a) and communicating the key message clearly: “*some education for the public […]*” (FG1 P9). A key point to communicate is that an elevated Lp(a) increases cardiovascular risk for an individual: “*I think actually, it’s useful [Lp(a) test] because then the patient know, oh my risk is actually higher.*” (FG1 P6).

To address skepticism and concerns about trustworthiness, educational materials for the general public and patients should be from relevant government agencies or institutions that are authoritative and non-commercial, such as the Ministry of Health, the Health Promotion Board, or the College of Family Physicians.

“*[…] printed, with Health Promotion Board’s logo; those are usually more convincing. […] rather than you print […] like Pfizer, you know, then that’s a bit of advertisement [Pfizer is one of the big four pharmaceutical companies globally].*” (FG1 P7)

“*[…] Health Promotion Board should come up with brochures on this thing [Lp(a) testing] now.*” (FG1 P5)

In addition, given the high prevalence of hypercholesterolaemia in Singapore, public education about cholesterol in general and familial hypercholesterolaemia would raise health literacy in this area.

“*I always tell my patients there are two sources of cholesterol. Not just from your food, your liver makes it too. So, if you have familial [hypercholesterolaemia], it’s just a thermostat set higher; so that they’ll make more [cholesterol]. […] so no matter how clean your diet is […] it’s gonna be at that level.*” (FG2 S8)

Participants cited the HPV test as a successful example of a government-led initiative. “*I mean, just like the HPV test previously. When I offered it to my patient like 10 years ago, they wouldn’t want. […] You tell them, I’m going to do this PAP smear and HPV, it costs you $200, they look at you, and they really think you are trying to make money. But ever since 2019, when the Ministry came out with the change, all my patients just accept it. Because it’s recognized. So, same [with] Lp(a).*” (FG1 P6)

Mass and social media would be effective ways for disseminating information/education to the general public: “*[…] the media is very important […] MOH definitely recognizes that.*” (FG1 P5), providing examples of public awareness campaigns during the SARS and COVID-19 pandemics.

##### 3.4.2.2 Subsidising Lp(a) tests

Cost-related discussions in both focus groups regarding patients’ reluctance to accept Lp(a) testing indicated that avoiding or minimizing out-of-pocket expenses would be an important strategy. Access to cheaper or subsidized Lp(a) tests is an enabler for both GPs (to initiate the test) and patients (to accept the test).

##### 3.4.2.3 Financial incentives for preventive health

Financial incentives may be useful in motivating the general public and patients to care for their health. For example, reducing certain types of national health insurance premiums.

“*Your cholesterol good, your diabetes good, your blood pressure good […] body fat good, 10% off [for each achievement]. […] age-appropriate physical fitness […] that is total 50%. You get 50% off Medishield Life.*” (FG2 S1)

### 3.5 LILAC-for-Lp(a) questionnaires

**Figure 2:**
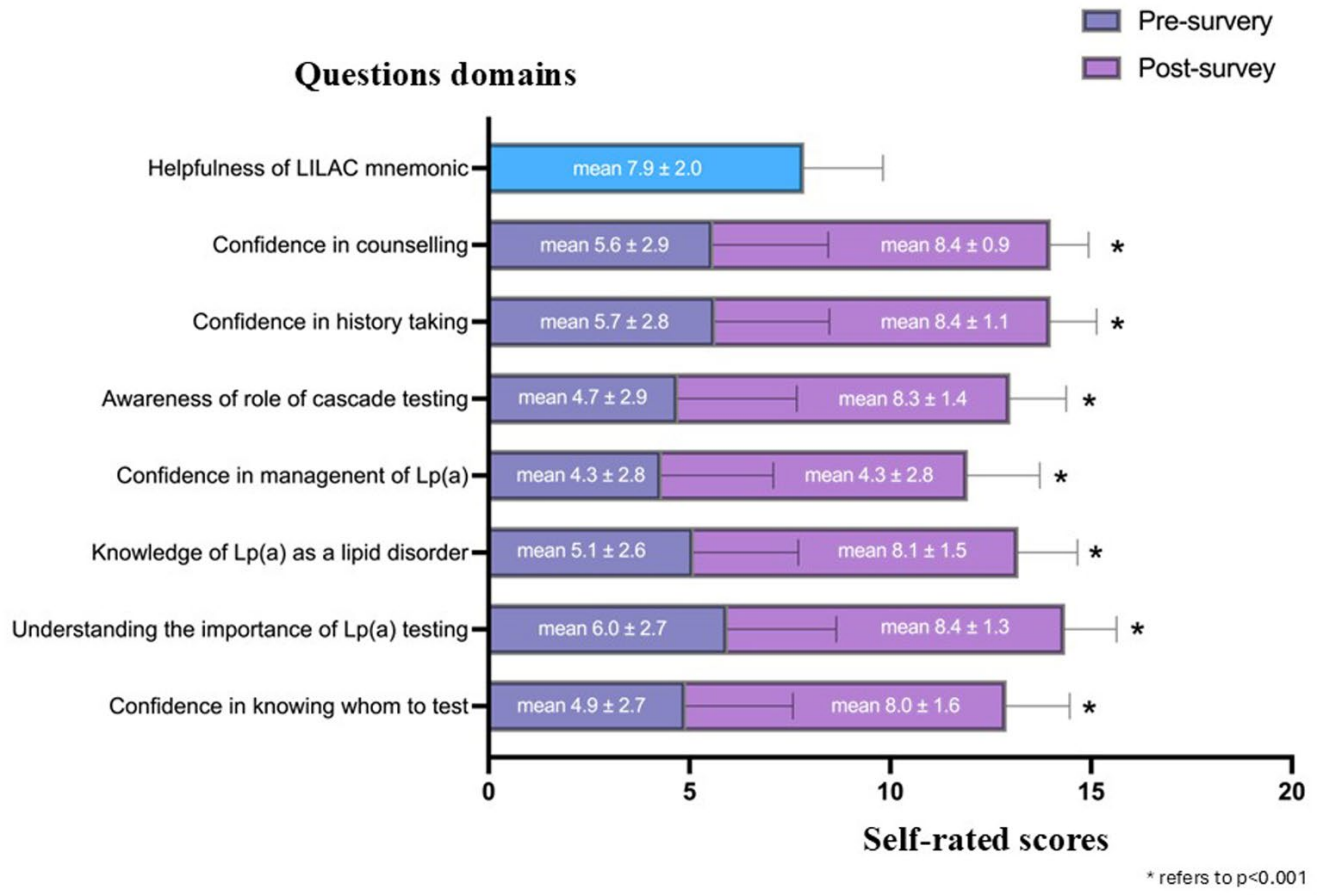
Responses to the questionnaires administered to the 50 GPs before and after the two short educational videos on Lp(a) are shown as stacked bars. The question about confidence in history-taking and counselling was asked only of 20 GPs who attended the in-person educational forum. The maximum score in each questioannaire (pre and post) is 10 points.

Figure 2 presents the results of the pre- and post-LILAC educational video questionnaires administered to 50 GPs. Comparison of the LILAC-centred education demonstrated a significant improvement following LILAC educational video in all domains assessed, including confidence in knowing testing indication (4.9 ± 2.6 vs. 8.0 ± 1.6, *p* < 0.001), importance of Lp(a) testing (6.0 ± 2.7 vs. 8.4 ± 1.3, *p* < 0.001), knowledge of Lp(a) (5.1 ± 2.6 vs. 8.1 ± 1.5, *p* < 0.001), and confidence in managing Lp(a) (4.3 ± 2.8 vs. 7.6 ± 1.8, *p* < 0.001). Similarly, there was significant improvement in awareness of the role of cascade testing (4.7 ± 2.9 vs. 8.3 ± 1.4, *p* < 0.001). Among the 20 GPs who attended the in-person educational forum and watched the LILAC educational videos, specific questions on confidence in history-taking and counselling were asked; significant improvements were observed in both areas: confidence in taking histories from patients with elevated Lp(a) (5.7 ± 2.8 vs. 8.4 ± 1.1, *p* < 0.001) and confidence in counselling patients (5.6 ± 2.9 vs. 8.4 ± 0.9, *p* < 0.001).

Before the Lp(a) educational forum, the pre-LILAC education questionnaire found that only 2 of the 20 (10%) actively practising GPs routinely tested Lp(a) in their clinical practice, indicating severe undertesting. In a multiple-choice question of why Lp(a) was not routinely tested, nine GPs did not test because of uncertainty about who should be tested, five GPs cited the lack of a clear management pathway, and two indicated that the cost was a barrier. One GP commented under the option ‘Other reasons, because the patients were unaware of Lp(a), additional time was required to counsel them on Lp(a) testing, posing a barrier to testing in a busy clinical practice.’ All GPs attending the educational forum responded that they would recommend LILAC educational videos to their colleagues.

**Figure 3:**
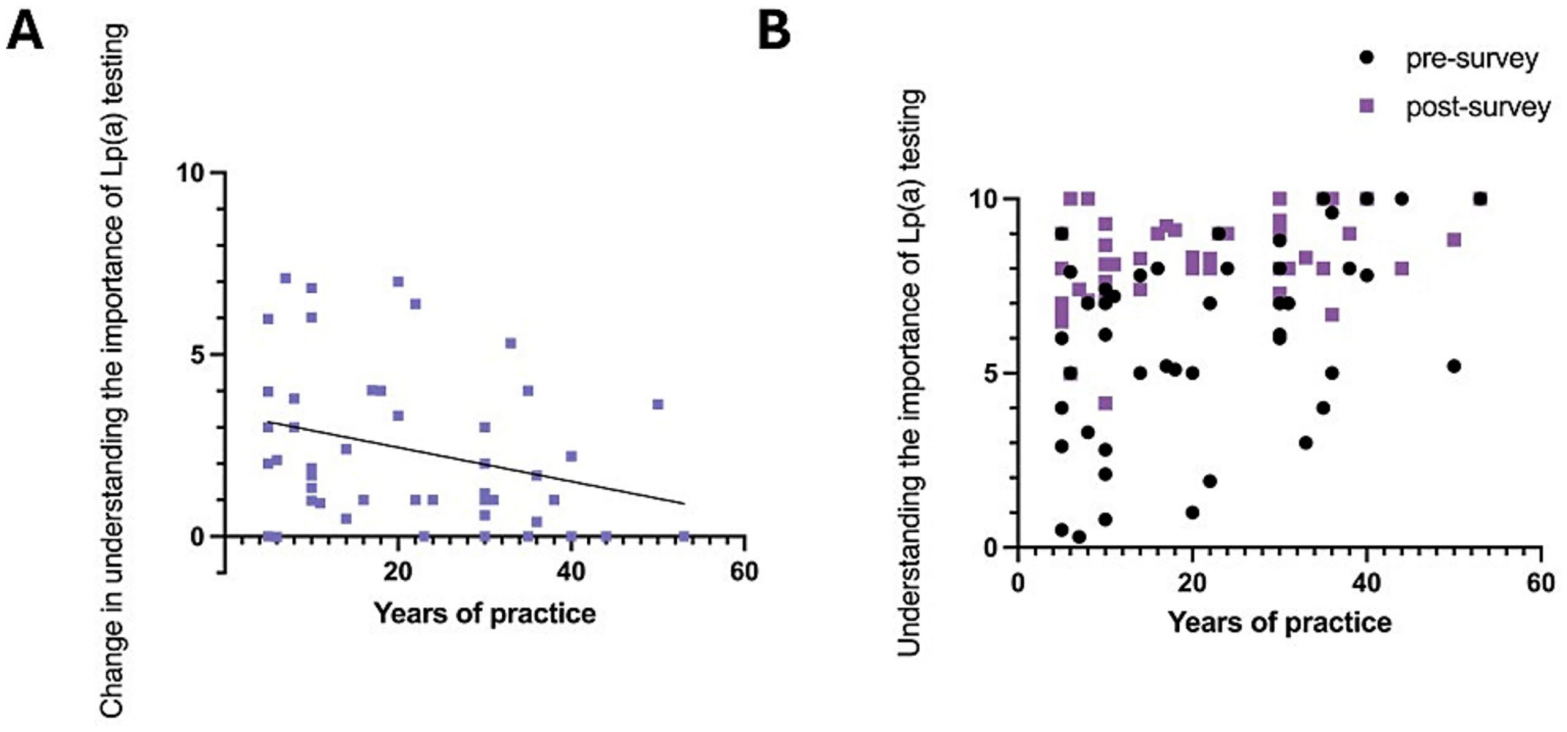
Correlation between years of working experience as a GP and the self-rating score of understanding the importance of Lp(a) testing **(A)**, and the scatter plot of scores in the pre- and post-video response to this question **(B)**, as shown.

Among the 50 respondents, 98% responded that the LILAC educational approach changed their perspective, making them view Lp(a) as an independent cardiovascular risk factor that can already be used in clinical practice. The respondents rated the helpfulness of the LILAC-for-Lp(a) mnemonic high (mean score = 7.9 ± 2.0). GPs with fewer years of experience were more likely to report that the educational videos had positively changed their perspectives on the importance of Lp(a) testing (*r* = −0.29, *p* = 0.043, Figure 3). However, there was no significant correlation between years of practice of GPs and other questions [e.g., confidence in managing Lp(a) and knowledge of Lp(a)].

When asked to specify what they liked about LILAC-for-Lp(a) education and how it could be improved, the GPs provided favourable reviews. Eleven GPs mentioned, “*LILAC was easy to remember and recall*.” Others mentioned “*excellent*,” “e*asy to remember and apply in the clinic*,” “*simple to remember and implement,” “clear and concise*,” and “*a catchy mnemonic*.”

Notable positive comments pertain to the structured and practical framework. The written feedback included: “A structured framework to remember how to manage Lp(a)”; “Clear and systematic way of management”; “It’s a useful mnemonic to make sure we know how to approach the problem and give the correct advice and treatment”; “recognizing the role of Lp(a) in cardiovascular risk assessment,” “understand its impact better,” “helps guide decision making especially for intermediate cardiovascular risk” and “A good flowchart.” Regarding how LILAC can be improved, some GPs recommended adding a statement, “Should include as a screen-for-life test,” and providing more nutritional advice.

## 4 Discussion

To our knowledge, this is the first qualitative study to investigate GPs’ perspectives on Lp(a) in Asia. The major barriers faced by GPs were the absence of a local consensus statement on Lp(a) and no ‘top-down approach’ by health policymakers to formalize Lp(a) testing. Other key barriers were the absence of an applicable workflow for primary care and inadequate training in managing elevated Lp(a) levels. Notably, similar insights were also elucidated in our qualitative research study of cardiology healthcare professionals, confirming a recurring theme of barriers (5). Furthermore, our study found that a structured, short educational programme using the LILAC framework positively changed GPs’ attitudes towards testing for and appropriately managing Lp(a). This was achieved by addressing misconceptions and reframing the mindset from ‘no actionable management for elevated Lp(a)’ to ‘yes, actionable’.

The factors governing patient selection for Lp(a) testing are in tension with the practical barriers encountered by GPs during testing (Themes 3 and 4). Practical barriers include unclear access to testing and the absence of region-adapted clinical guidelines recognized by local medical governing bodies. However, despite these barriers, GPs agreed that Lp(a) should be included in routine cardiovascular risk assessments. Appropriate strategies put forward by GPs included providing effective education and training in lipid management, including Lp(a), as well as prioritizing local consensus statements or guidelines recognized by the local governing body (e.g., Singapore’s Ministry of Health). The focus group discussions revealed unanimity that without local policy changes and mass training, GPs are likely to continue managing common hypercholesterolaemia, familial hypercholesterolaemia, and elevated Lp(a) as they have in the past, rather than according to the latest international guidance or expert opinions (Theme 1). Thus, a local expert consensus statement on which patients should undergo Lp(a) testing, cascade testing, and treatment pathways, as well as the subsidized cost of Lp(a) testing via existing billing mechanisms (e.g., Medisave or CHAS), is required.

As shown by the focus group discussions with GPs and past research (5), reticence towards Lp(a) testing in practice stemmed from knowledge gaps, highlighting the need for targeted education and training strategies (Theme 3). Knowledge gaps were in multiple aspects, including Lp(a) as a hypercholesterolaemia condition and the interpretation of the results. The LILAC framework was developed to directly address such misperceptions, and its effectiveness was subsequently reflected in our quantitative findings. Prior to LILAC education, the majority of GPs had a tentative approach to Lp(a) due to perceived patient attitudes towards genetic lipid disorders. Although GPs’ patient selection for testing Lp(a) was partially informed by clinical indications and rationale, it was clearly confounded by the social concerns of their patients’ presumed unwillingness to being diagnosed with a genetic condition (Theme 2) and the presumed high cost of testing (a misconception). Patient advocacy groups disagree with the notion that people would be unwilling to be tested for Lp(a), a simple, non-fasting blood test that does not involve DNA (4). When the cost of testing of Lp(a) blood test locally (USD $30–75, or SGD $30–120) was clarified to the study participants, they changed their minds and unanimously agreed that the cost was reasonable in the local context (5, 9). Two other notable misconceptions were that (i) Lp(a) could replace other testing modalities (e.g., CT calcium test) and (ii) there were no actionable strategies for elevated Lp(a). However, studies suggest the combination of CT coronary artery calcium score with Lp(a) together is useful for risk stratification and thus guiding treatment intensification (20). Using the LILAC concept, the GPs and healthcare professionals, including nurses and allied health professionals, learned that there are indeed multiple actionable strategies, as illustrated by its 5 principles (14). The LILAC approach summarises evidence-based expert recommendations and principles of management, highlighting the importance of overall cardiovascular risk factor management, such as with further risk stratification tools [e.g., CT calcium score, family history, CRP, Lp(a)], and adoption of a healthy lifestyle, lowering LDL-cholesterol as the key methods to managing cardiovascular risk conferred by elevated Lp(a) and cascade testing of family members when the index case has very high Lp(a) (2, 11, 14). These two important misconceptions were addressed in the two LILAC educational videos, likely explaining the significant improvement in GPs’ confidence in testing and management in the post-video questionnaires.

Multiple studies, including ours, have highlighted the need for better education and training programs for doctors to manage patients with elevated Lp(a). This is despite the recent widespread coverage of Lp(a) at medical conferences on atherosclerotic cardiovascular diseases. Recently published studies, both locally and worldwide, have shown that major gaps in knowledge and practices exist and that there is low confidence in managing Lp(a), even among specialists in cardiovascular disease management (7–9, 14, 21, 22). The Interaspire study of 245 physicians from 14 countries, 28% of whom were GPs, reported that 43% had local access to Lp(a) testing. Out of those with access to Lp(a) testing, only half routinely tested Lp(a) in all patients with coronary artery disease, whereas one-third were unsure of Lp(a) interpretation (21). The short educational videos on #LILAC-for-Lp(a) via online platforms and webinars may be a feasible option for continuing medical education for existing GPs and other primary care healthcare professionals and can be adapted to suit local practices (14).

A cost-effectiveness analysis from UK and Australian primary prevention healthcare perspectives found that Lp(a) testing is cost-saving, with an estimated saving of $85 per person in Australia and £263 per person in the UK (23). Hitherto, there have been no cost-effectiveness studies in Asian settings. Future studies should include cost-effectiveness and feasibility analyses, as well as randomized trials to evaluate the most effective and sustainable implementation strategies in primary and secondary cardiovascular disease prevention settings. Lessons could be learned from nurse-led cascade screening programs, such as for familial hypercholesterolaemia (24). These studies need to involve primary care, given the common condition of hypercholesterolaemia with a strong genetic predisposition (11).

This study has several limitations. The GPs who participated in these focus groups were those who signed up for a continuing medical education forum on Lp(a). They may be more motivated and lipid-focused than the broader GP population, which could introduce self-selection bias. Hence, their views may not be representative of GPs who did not participate. Only two focus groups were conducted. Hence, although data saturation was achieved, thematic saturation may not have been achieved. While focus groups are an established qualitative method, the quality of the discussion, and hence the richness of the data generated, depends on the skill of the facilitators and participants’ engagement. This was mitigated by involving external expert focus group facilitators in the focus groups. Study participants were GPs in private practice and do not represent the views of primary care doctors working in government-subsidized clinics (i.e., polyclinics), which use a different billing system. However, as part of the national preventive health programme called Healthier SG, GP workflows, and management are aligned with those of primary care doctors in polyclinics (25). There were only a small number of participants in the pre- and post-questionnaires, and this will be followed by a larger multisite educational study (clinicaltrials.gov NCT07177612).

A strength of this study was its reach of more than 2000 GPs across the country via GP network leaders and emails. Study participants included both genders, with varying degrees of work experience, and were from different parts of the country. In addition, Singapore’s GP practice setup is similar to that in many parts of Asia; thus, our findings are likely to provide relatable and applicable insights elsewhere.

## 5 Conclusion

This study of actively practising GPs identified key barriers and enablers to Lp(a) testing in Singapore’s primary healthcare system. The key barriers were major knowledge and awareness gaps about the clinical importance of Lp(a) testing and management and a unanimous call for national initiatives to integrate Lp(a) into a systematic workflow. A starting point is the recognition from public health and primary care that Lp(a) is a risk factor for atherosclerotic cardiovascular disease. The LILAC-themed educational lectures were useful in shifting GPs’ mindsets to test and manage this common, chronic, inherited hypercholesterolaemia.

## Data Availability

The original contributions presented in the study are included in the article/Supplementary material, further inquiries can be directed to the corresponding author.
