# Supplementary material for: A multi-method study on GPs’ perspectives on Lipoprotein(a) as an actionable cardiovascular risk factor
Source: Front Med (Lausanne). 2026 Jun 12;13:1859808. doi: 10.3389/fmed.2026.1859808 (PMC13303360; doi:10.3389/fmed.2026.1859808)
Supplement: Supplementary file 1 [file Data_Sheet_1.PDF]

APPENDIX

THEME 1. CURRENT CONTEXT

**CATEGORY: GP Current Practice — Hyperlipidemia & Familial Hyperlipidemia**		
Sub-category: Identification and treatment of (familial) hyperlipidemia		
Code<br>[Description]	Sub-code (if any)<br>[Description]	Representative quotes from participants<br>(quotes may appear in more than one code or sub-code if relevant)
Identification of (familial) hyperlipidemia<br><br>[GPs state how patients with hyperlipidemia or familial hyperlipidemia are identified]	NA	FG1 P9: "[…] we see a lot of cases that come in [for] health screening […] and we pick them up incidentally. Although [if] they already have concomitant hypertension and diabetes […] we always do [lipid tests] as a routine test for them."
Prevalence of (familial) hyperlipidemia<br><br>[GPs perceived prevalence of hyperlipidemia and familial hyperlipidemia]	NA	FG2 S3: "In our local GP clinic, we don't really see a lot of familial hyperlipidemia unless they tell us"<br><br>FG2 S4: "[…] most cases, actually not really familial la. I mean, in my opinion, because you don't know the cholesterol level of the parents."<br><br>FG2 S8: "I have seen a lot of hyper -- familial hyperlipidemia, not -- not like labeled, but you you can see the pattern because we take a very detailed family history. So, we see that, okay, more than three or four males are all, you know, dying early from acute MI. So -- and cholesterol, you know, how many relatives have so you already kind of can tell, okay, this is probably familial."
Treatment of (familial) hyperlipidemia<br><br>[GPs state how they currently treat patients with hyperlipidemia or familial hyperlipidemia]	Treatment based on established guidelines<br><br>[GPs treat hyperlipidemia based on established guidelines]	FG1 P9: "[…] we base our treatment on guidelines […] recognized in Singapore and internationally. So it's easy [to decide on treatment] because we follow the guideline."
	Aggressive treatment<br><br>[Aggressive treatment of hyperlipidemia whether it is familial hyperlipidemia or not, especially for younger patients and patients with pertinent personal or family history]	FG2 S2: "The academic side, there will be different kinds of hereditary familial hyperlipidemia, but on our GP side, as far as I'm concerned, I'm just trying to bring it [LDL] down […] I will treat aggressively […] my aim is […] to bring down the LDL […] whether it is familial or not […] is academic. […] young people with cardiovascular diseases, then the urgency is there."

		FG2 S8: "[after assessing family history] so you already can tell, this is probably familial, and they probably will not respond to lifestyle modification alone. Another set of people that I find with high LDL is hepatitis B carriers. Their livers are damaged […] so their lipids are high […] go straight [treat with] into statin."
	Current treatment protocols may be outdated<br><br>[GPs are using locally available treatment protocols which do not factor in Lp(a); thus, may be outdated]	FG2 S5: "But we are supposed to follow the protocol, this is what they set out for us to do. […] maybe 10 to 20 years later, then they will say, oh, based on this [Lp(a) test], yes, you probably at a higher risk. But now at this moment of time it [the protocol] is based on past 20 years' history kind of a data. May not – may not be [accurate]."
	Current treatment protocols seem to conflict with GP clinical judgement<br><br>[GPs are puzzled that current HealthierSG protocols seem to conflict with their clinical judgement to start patients on lipid-lowering medication]<br><br>Note: high LDL alone without other risk factors is not usually an indication for statin therapy, unless LDL ≥ 190mg/dL	FG2 S5: "[…] as a GP right, so usually we do some health screening for them, right? […] nowadays we have this HSG [HealthierSG] so we do a lot of screen4life then. […] we do the usual cholesterol check […]. So, the next steps, you look at the protocol la, […] we can calculate the risk factors, based on the age, the sex, and whether they're smoking or not whatever. So, we just use the calculator. If the calculator says uh high risk then we start them on a statin la. But unfortunately, I think for the past few months, I check the calculator if the LDL is high, let's say you are 180 or 200 but they don't have other risk factors, the calculation shows that still considered as low risk. So, there is no indication you know, to start the statin … even though like wah 180 milligram, almost uh 200, 190 LDL. The calculation is low risk you know. So, they say low risk less than 5%. There's no indication for me to start, you know, for the medications. […] So, I was wondering why — what's wrong with the calculator sometimes. So, I mean, in absolute numbers [LDL] high. But there's no indication to start [statin].

Managing a hypothetical patient with elevated Lp(a) after hospital discharge<br><br>[How GPs would manage a hypothetical patient with elevated Lp(a) after hospital discharge]	NA	FG1 P9: "Usually the specialist will start treatment, like, maybe increase the statin […]. Then you just follow up with the recommendations from the specialist. Carry on the medicine and then you monitor the LDL; and of course, you have to be aware that the LDL is supposed to be lower than normal, because you have an Lp(a) positive [patient]. […] I'm sure they also have a yearly check up with their cardiologists."
Sub-category: Cascade testing		
Cascade testing for familial hyperlipidemia<br><br>[What GPs do regarding cascade testing for familial hyperlipidemia]	NA	FG1 P7: "The guideline board [..] for the hyperlipid treatment is pretty clear right now […] they actually do say that we have to refer the familial hyperlipidemia patient to the hospital for family checking"
Acceptability of cascade testing<br><br>[GP's perception on patient acceptability of Lp(a) cascade testing]	NA	FG1 P9: "I think it depends on individual families, how they value their health, you know. Do they see this as important to them or not. […] I mean, you can tell the patient to invite the family members, but of course, you also must tell them the risk of the insurance […] if positive [elevated Lp(a)]. […] let them decide; whether […] health is more important or insurance premium is more important"
Likelihood of elevated Lp(a) detected through cascade testing<br><br>[The likelihood of elevated Lp(a) being detected in family members through cascade testing]	NA	FG1 P2: "I want to know […] what is the chance that the patient's brother or sister will have a positive [elevated] Lp(a) also, and the second degree relative. […] if you tell me that even among siblings it's only 30%, then I would be a bit hesitant to ask […] family members to come for screening."
Unwarranted cascade testing?<br><br>[Cascade testing may be unwarranted if clinical evidence shows sufficiently high degree of heritability]	NA	FG1 P5: "[…] let's say that penetrance is as high as 80, 90%. Why need to test, if one of them is already high [elevated Lp(a)]? You have a high chance of having that."

APPENDIX

Sub-category: Triggers for referral to specialist		
Uncontrolled hyperlipidemia<br><br>[Uncontrolled hyperlipidemia, especially in patients with other co-morbidities]	NA	FG2 S1: "If you can't [manage/control patient's hyperlipidemia], it's still high, high, high; then you better let go [refer to specialist]."
Multiple co-morbidities and risk factors<br><br>[Patients with multiple co-morbidities and risk factors that confer a high risk of cardiovascular events]	NA	FG2 S8: "I think, also if it is multiple comorbidities like, diabetes, you know, uncontrollable hypertension, then hyperlipidemia; this guy, smoking, man [male], Indian, wah. Okay, better go to cardiologist sooner rather than later."
Elevated Lp(a) not a trigger for referral to specialist<br><br>[GPs feel capable of managing patients with elevated Lp(a)]	NA	FG1 P7: "I don't think the Lp(a) will cause us to refer it out [to specialist] because we know it is a risk factor, just assume it is as though added on a diabetic. It's a non-diabetic now with Lp(a) positive, so we treat it like a diabetic […] work towards this 30 [mg/dL of LDL] with or without medicine, then medicine has diabetic side effect, but it is not that high diabetic;" and "[…] because of Lp(a) positive and hence referred off to the cardiologist, actually not really necessary. Because there's, well, no treatment right now, so we don't need a cardiologist to treat."
Sub-category: Loss of patient post-referral		
One-way referral loop<br><br>[GPs lose their patients post-referral to specialist]	"They don't come back"<br><br>[Patients remain with the specialist]	FG2 S3: "Once we refer, they stay with the specialist. They don't come back."
	"They are referred to polyclinic"<br><br>[Patients referred to the polyclinic instead of to their GP]	FG2 S3: "Normally, the specialists don't refer back to us. […] when they discharge the patient go to polyclinic, they just skip the GP when we are the first one to refer to them."
	"They choose polyclinic"<br><br>[Patients choose to go to the polyclinic instead of returning to their GP, due to cost of medications]	FG2 S2: "[…] specialist will give the patient a choice of going back to the family doctor or the polyclinic."<br><br>FG2 S6: "[…] chronic meds are cheaper there [polyclinic]"

APPENDIX

CATEGORY: Patient Attitudes — Hyperlipidemia		
Sub-category: Delay and denial		
Code<br>[Description]	Sub-code (if any)<br>[Description]	Representative quotes from participants (quotes may appear in more than one code or sub-code if relevant)
Delay being diagnosed and treated<br><br>[Patients delay being diagnosed with hyperlipidemia and reluctant to start lipid-lowering medications without attempting lifestyle changes first]	NA	FG2 S6: "[…] yes, the lipid levels are high. Then they just say, 'oh, give me three months' and they try to […] do some lifestyle changes, you know? But more often than not, we don't see them coming back. […] they don't want to put a stamp at such a young age that they have high cholesterol levels."
Denial — Repeat cholesterol test<br><br>[Patients request to repeat cholesterol test to further convince themselves or to delay treatment]	NA	FG2 S8: "They're resistant [to treatment] in the first time; so you say, okay, come back three months later, check again"<br><br>FG2 S4: "That ties in with my observation that over the years ah, a lot of patients, oh ok its high [cholesterol] you know, […] 6 months' time we do another one. And almost invariably about the same."
Reluctant to start lipid-lowering medications<br><br>[Patients are reluctant to start lipid-lowering medications]	Concerned about side effects<br><br>[Patients are concerned about side effects of lipid-lowering medications]	FG2 S7: "[patients] read about muscle ache and all that, you know. […] they blame the -- every muscle ache on the statins, right?"
	Concerned about lifelong usage<br><br>[Patients are concerned about lifelong usage which puts them off starting lipid-lowering medications]	FG2 S7: "[…] they [patients] are afraid that that they have to take [statins] forever -- but it's true."<br><br>FG2 S3: "[…] for example I start [them] on statin, let's say the patient finally agree, most of the time they are resistant to continue taking, we know that they should."

Reluctant to continue lipid-lowering medications<br><br>[Patients are reluctant to continue lipid-lowering medications; GPs use n=1 experiments to convince them]	NA	FG2 S3: "for example I start [patient] on statin, let's say the patient finally agree. Most of the time they are resistant to continue taking, we know that they should, so always I give them some hope, okay, we try six months of medicine, we stop, because you want to stop right; we do a blood test, show that it's gone down right, 50%; we are aim for 50% reduction. Then [for] six months, you carry on your whatever lifestyle, your diet, or whatever you believe ah any herbal medicine. Six months later, we check [again] most of my [patients] […], went up back […] take medicine, went down 50%, then stop medicine. Then I say these six months you do whatever you want, then that six months later one year from the first one [test] you went up almost the same as the first […] they say 'huh that means I cannot stop [statin]'. Then, ya, now you prove to yourself cannot stop."
Sub-category: Not a serious problem, unless…		
Hyperlipidemia is not a serious problem<br><br>[Patients without relatively recent first-hand experience (either themselves or a family member) of cardiovascular events, do not consider hyperlipidemia to be a serious problem]	It's not cancer<br><br>[Hyperlipidemia is not as serious as cancer]	FG2 S3: "They don't think […] having [high] cholesterol in the family is like having cancer. […] they wouldn't act on it [high cholesterol] and be serious about it."
	"Patients are not bothered by high cholesterol"<br><br>[Explanations by GPs were insufficient to motivate patients to take hyperlipidemia seriously]	FG2 S6: "I always have to like, give them analogy, can you imagine coating, like oil in your garden hose? And then it doesn't -- you cannot u-turn that process, especially in your blood vessels and all […] but a lot of times they – they are not – they are not too bothered by it."<br><br>FG2 S8: "I've emphasized -- the possibility of a heart attack, because, um that is what it leads to. And if they are not convinced, I will tell them to do CT calcium score."

	Unless witnessed — themselves or family member<br><br>[No first-hand experience (either themselves or a family member) of cardiovascular events]	FG2 S7: "Patients who have family members [with] cardio events […] they are a bit scared and they would go for the screening. […] if they have seen their parents in CCU [coronary care unit], then they will really go for that [screening] you know."
	Unless relatively recent<br><br>[Family member with cardiovascular event which occurred long ago]	FG2 S7: "However, if they, the parents have passed away, years ago [patients may or may not go for screening] actually they will, sometimes they will actually. And then they [are] diagnosed with um high LDL or low HDL."

THEME 2. PATIENT SELECTION FOR Lp(a) TESTING

**CATEGORY: Clinical Rationale**		
Sub-category: To replace calcium scoring		
Code<br>[Description]	Sub-code (if any)<br>[Description]	Representative quotes from participants<br>(quotes may appear in more than one code or sub-code if relevant)
Replace expensive calcium scoring<br><br>[Lp(a) test offered instead of more expensive tests such as calcium scoring]	NA	FG1 P7: "I don't do [Lp(a)] for everybody. I do for those who are […] borderline high cholesterol […] whether you want to do calcium scoring to identify whether they are very high risk. But to do calcium scoring means we have to refer […] costs hundreds of dollars. So, I use Lp(a) [test] as a so-called replacement."
Sub-category: Younger patients		
Younger patients with personal or family history<br><br>[Younger patients with personal or family history of cardiovascular disease]	NA	FG2 S3: "[…] family history wise, some very young person with heart events, then we will encourage the whole family to go for screening. […] if it is themselves having a heart attack below 50 [years old], then of course also ask him to ask family members to screen."

		FG2 S2: "if the risk factors in the family is high there is some -- some young people with cardiovascular diseases then it -- the urgency is there."
Sub-category: Timing and need		
Individualize the timing<br><br>[Time the Lp(a) test on a case-by-case basis, i.e. after addressing the patient's known risky behaviours]	NA	FG1 P1: "[…] let's say the guy has […] terrible cholesterol […] then you don't need to do the Lp(a) first. […] this would be an unnecessary supplementary test. He's already smoking 20 sticks and all that, so […] it's not gonna be much of a difference."
Justify the need<br><br>[Justify the need for Lp(a) test on a case-by-case basis]	Unwarranted for patients with known high risk?<br><br>[Lp(a) test may be unwarranted for patients known to be at high risk for cardiovascular events]	FG1 P1: "[…] if you already have the whole Christmas tree full of other risk factors already […] and his dad died at 30; then there's no need to do Lp(a) [test]. You know that he's very high risk already."
	Unwarranted for patients with optimized treatment?<br><br>[Lp(a) test may be unwarranted for patients who have achieved treatment goals]	FG1 P7: "if the patient has a- I mean, his target is already 55. lower than 55, already has heart attack as smoker. Then what does adding on an extra risk factor in this target 55, how low can you go? I mean, it wouldn't help, then I will not do."
	To ascertain cardiovascular risk profile<br><br>[Lp(a) test is useful for ascertaining a patient's cardiovascular risk profile]	FG1 P7: "I don't do [Lp(a) test] for everybody, I do for those who are […] those who are a little borderline high cholesterol."
Lp(a) testing may reduce clinical inertia in intensifying treatment<br><br>[An elevated Lp(a) result may encourage GPs to treat more aggressively]	NA	FG1 P9: "now the new thinking is you have to adapt to the new knowledge and then use it to for the patient's benefit. Because you cannot just say, ah okay lah, this [Lp(a)] is not important, then just forget it, then you are not helping the patient."<br><br>FG1 P2: "if it's positive [elevated Lp(a)], we may want to be very, very strict with your LDL control. Even go by guidelines, this seems to be normal, no need to

		start statin, but you have a higher risk and based on the studies, quote if you can, you know, those evidence that you mentioned, right? Show them 10 years risks, 50% increase mortality, for example, in how many years, then it would be convincing for a young patient who actually didn't think much about that casual statement.”
Lp(a) testing may motivate reluctant or undecided patients to start treatment<br><br>[An elevated Lp(a) test may motivate reluctant or undecided patients to start lipid-lowering treatment]	NA	FG1 P9: “You actually encourage your patient to start statins rather than delay.”<br><br>FG1 P6: “I think actually it’s useful because then the patient knows, oh my risk is actually higher […] (Let’s say my LDL?) is 140, but with Lp(a) then my real risk is higher.”<br><br>FG2 S1: “So the patient actually has the impetus to do better [meet treatment goals], you can't say you don't care. Because if I come for the study [Lp(a) test], then the insurance will say you are high risk you know because you got diabetes [risk factor], something like that. […] So, that guy [patient] cannot forget that he’s not well in that sense because there are implications as to the assessment. And now that he knows it, everyone knows it as in like, he can't deny.”
Lp(a) testing for everyone?<br><br>[What GPs think about Lp(a) testing]	NA	FG1 P7: “[…] at this moment of time Lp(a) is probably not for everybody like you do for the aunties, they are 70s, 80s.”

APPENDIX

CATEGORY: Economic Rationale		
Sub-category: Affordability		
Code<br>[Description]	Sub-code (if any)<br>[Description]	Representative quotes from participants (quotes may appear in more than one code or sub-code if relevant)
Cost of Lp(a) test<br><br>[Whether patients can afford and/or willing to pay for Lp(a) test]	Unable to pay<br><br>[Poorer patients are unable to afford Lp(a) test unless subsidized]	FG1 P3: "[…] my patients do not want to pay the extra money […] I actually work at heartland. So, they [patients] tell me […] whatever can be deducted, you know, from CHAS. By the time you do a blood test, […] the medication, already that is the [CHAS] limit. You hit the [CHAS] limit. I can't really do [Lp(a) test]."<br><br>FG2 S5: "[…] we are not doing it [Lp(a) test]. It's not indicated at all. […] it's not covered by Screen4Life [national health screening program rebranded to HealthierSG]."
	Unwilling to pay<br><br>[Patients may be able to afford Lp(a) test but unwilling to pay]	FG1 P3: "Company [panel clinic], [patient] wasn't very keen […] because it's not part of our official package [of tests]. So, [patient] refuses to top up extra [for the Lp(a) test]. So, it's not easy at this moment."<br><br>FG1 P1, P3: "because it's not free."
	Lp(a) test not bundled with lipid panel<br><br>[Lp(a) test not bundled with lipid panel or other test packages; thus, incurring extra cost]	FG1 P2: "[…] it's not under the standard lipid panel."<br><br>FG1 P3: "Company [panel clinic], [patient] wasn't very keen […] because it's not part of our official package [of tests]. So, [patient] refuses to top up extra [for the Lp(a) test]. So, it's not easy at this moment."
	Management of other comorbidities prioritized<br><br>[Management of other comorbidities are prioritized over Lp(a) testing for patients on CHAS due to limited claimable subsidy]	FG1 P5: "I have not ordered the test […] those who are on CHAS, even if they are on a blue CHAS, and even if they have multiple illnesses, they can only claim up of $95 per visit [maximum claim]. If this test itself cost $55, it is very, very hard to convince them to do that."

	Proactive patients who can afford the Lp(a) test<br><br>[GPs may offer the Lp(a) test to proactive patients who can afford it; these are usually better educated patients from higher socio-economic classes]	FG1 P7: "But the very educated ones would choose to do my $400-$500 health screen, and they ask me […] 'anything else you think I should do?' […] Let's do an Lp(a)."
Sub-category: Implications on health insurance		
"Insurability"<br><br>[Whether the patient/family members would still be eligible for health insurance if found to have elevated Lp(a)]	Impact on eligibility for health insurance<br><br>[GPs discuss the potential impact on eligibility for health insurance for patients (and their family members) if found to have elevated Lp(a)]	FG1 P8: "Because you may just subject *[sic]* your patient's children from buying insurance in future. Because if they are all found [to have elevated Lp(a)], then the declaration problem. So, do you really want to do the test for them?"
	No/minimal adverse impact on eligibility for health insurance<br><br>[Elevated Lp(a) may not have adverse effects on eligibility for health insurance (patient or their family members)]	FG1 P2: "[…] insurance companies they also have their panel of doctors; if your Lp(a) is not a proven conclusively to notch it up by a lot, the panel of doctors in the insurance company also know, right? Yeah, so I don't think you are jeopardizing the patient's chance at all."<br><br>FG1 P7: "So far, no insurance report ask for if family have anything."<br><br>FG1 P9: "Insurance staff don't routinely ask for Lp(a) status, right?"
Insurance premiums<br><br>[An elevated Lp(a) may cause patients/family members to incur higher health insurance premiums]	NA	FG1 P1: "So, the [elevated] Lp(a), […] they will insure you at a higher premium."

THEME 3. WHAT PREVENTS & ENABLES GPs TO INITIATE Lp(a) TESTING

**CATEGORY: GP Barriers for Initiating Lp(a) Testing**		
Sub-category: Knowledge gap		
Code<br>[Description]	Sub-code (if any)<br>[Description]	Representative quotes from participants (quotes may appear in more than one code or sub-code if relevant)
Awareness<br><br>[GPs were not aware or only recently aware of Lp(a) for cardiovascular risk assessment]	Unaware of Lp(a)<br><br>[GPs were not aware of Lp(a)]	FG1 P9: “So, this is something new for us doctors who graduated maybe 20, 30 years ago. We’ve never even heard of Lp(a).”<br><br>FG2 S7: “Super blind spot, super blind spot.”
	Newly aware of Lp(a)<br><br>[GPs were only recently aware of Lp(a)]	FG1 P7: “I only learned about it during the recent CME at [hospital redacted], […] saying that everybody, once in a lifetime should do a Lp(a), to see where you stand and that got me interested. […] So, I start doing Lp(a) probably about a month ago?”
	(Theoretically) Covered in medical curriculum but not applied<br><br>[Some GPs reported that Lp(a) was covered in medical curriculum theoretically but imparted no practical skills to manage patients]	FG2 S3: “It's not necessary [Lp(a) testing] um we learn it during our medical school but we don’t actually apply it, so even if you give me a number I do not know how to interpret it, so I don’t do it.”<br><br>FG2 S2: “So, when we doing chemical pathology, in ‘92, the chemical pathologist giving the lecture they were already talking about Lp(a) already. […] 33 years ago already, it was a standard chapter in the chemical pathology.”
Interpretation of Lp(a) results<br><br>[Being unsure of how to interpret and use Lp(a) results deter GPs from ordering the test]	NA	FG2 S3: “[…] so even if you give me a number, I do not know how to interpret it. So, I don’t do it [Lp(a) test].

APPENDIX

Sub-category: Lp(a) test not accessible		
Lp(a) not easily available<br><br>[GPs perceived that Lp(a) test is not easily available from pathology labs]	NA	FG1 P5: "No, because the test is difficult to get […] it's not readily available previously."
Lp(a) should be part of cardiovascular risk assessment<br><br>[GPs felt that Lp(a) should become a routine part of cardiovascular risk assessment]	NA	FG1 P6: "[…] I would definitely be in favor, it's just that the cost comes in. I was actually thinking, you know, we use the Framingham scoring for our patient to risk stratify. Actually, they should include Lp(a) […] if you have Lp(a) positive, you have one more mark, right? That would come in quite easily. Then you increase your risk for heart attack."<br><br>FG1 P9: "Yes, unanimous [agree]."
Sub-category: (Lack) Guidelines		
Lack of MOH guidelines for Lp(a)<br><br>[There are no officially sanctioned guidelines about Lp(a) from MOH]	No clinical pathway for testing and management<br><br>[No MOH clinical guidelines/pathway for Lp(a) testing and management]	FG1 P4: "[…] the Ministry hasn't formalized [guidelines]. […] You don't have anything [treatment pathway], […], you frighten the patient and what you gonna do? So, we [general practitioners] can't do anything about it."
	GPs unwilling to be "grassroots" advocate for Lp(a) testing<br><br>[GPs feel that Lp(a) testing should be "top down" not "bottom up"; hence, unwilling to be advocates without MOH guidelines to fall back on]	FG2 S2: "As long as Lp(a) not in the main MOH ACE guideline [I won't test/advocate] – it's not a bottom up grassroot movement. It has to be very top down. You've got to convince the powers that be and not grassroot fodders like us."<br><br>FG2 S1: "[…] the money is from them [MOH], the KPI is from them; so, you ask us [general practitioners] also no point."
	Public health agenda not focused on hyperlipidemia	FG2 S1: "that's [diabetes] where the war is what. Then there is a salt war. I think the Minister that time hasn't come to cholesterol yet. So, it may be a bit premature,

	[The lack of Lp(a) guidelines underscores GPs' perception that MOH is not focused on hyperlipidemia specifically]	but see how it goes"; "you go supermarket is all diabetic based what […] So, the Lp(a) is still long way."
	Robust evidence about Lp(a) testing will spur MOH to act<br><br>[GPs perceived that MOH will act swiftly if convincing evidence about Lp(a) testing is available, emphasizing the "top down" approach and their argument that GPs should not be "bottom up" advocates for Lp(a) testing]	FG1 P2: "Depends on the significance of the risk, right? If the risk is very, very low, then everybody will just dilly dally right, there's no real urgency, right? But if one day a study comes out that shows you that the risk is very, very high, then we [general practitioners] don't have to do anything [don't have to advocate]. […] it will automatically get done [directives from Ministry of Health], right? There will be mass info to the public."
	MOH should be made aware of robust evidence about Lp(a) testing<br><br>[GPs perceived that MOH is not aware of Lp(a)'s importance despite robust evidence being available]	FG1 P6: "[…] there is already quite a lot of studies done [about Lp(a)]. But I think it hasn't gotten up to the upstairs people [Ministry of Health], who are unfortunately […] not in this field [although] they are medical doctors."
	Lp(a) test "not recognized"<br><br>[Current MOH guidelines do not include Lp(a); thus, GPs question the legitimacy of ordering Lp(a) test as it is "not recognized" by MOH]	FG1 P7: "So, once you say that polyclinics can do [Lp(a) test] it means that upstairs recognize it; means that it is subsidized. […] Yeah, I dare not even bill Medisave because it [Lp(a) test] is not recognized."
	Lp(a) test "not indicated"<br><br>[Current HealthierSG protocols do not include Lp(a) testing; thus, GPs perceived that Lp(a) test is not needed]	FG2 S5: "[…] we are not doing it [Lp(a) test]. It's not indicated at all. […] it's not covered by Screen4Life [national health screening program rebranded to HealthierSG]."
Anxiety about governance and audits<br><br>[GPs were anxious about governance and being audited if they acted without clear MOH guidelines	NA	FG1 P7: "[…] and now with HealthierSG, they [MOH] are so sticky about step one to two and three. It is like following a real textbook […]"

about Lp(a) testing, citing an example from HealthierSG]		FG2 S5: "If you don't follow, they will penalize you."<br><br>FG1 P7: "[…] you can't claim on CHAS blue doing such a funny test [Lp(a)], you would be audited out."
Anxiety about medico-legal implications<br><br>[GPs were anxious about medico-legal implications if they acted without clear MOH guidelines about Lp(a) testing, citing potential litigation by patients]	NA	FG1 P4: "But if they [MOH] never say anything [no clinical guidelines] and you do it [Lp(a) test]; then the patient say 'oh hey, […] take statin may cause diabetes.'"<br><br>FG1 P8: "[…] if you do the test for them, even you give them free, the Lp(a), and later found to be positive, they can sue you in future. So, be very careful doing it because it's close, akin to, genetic testing."
Dissonance between guidelines<br><br>[GPs expressed confusion due to dissonance between guidelines]	Dissonance between international guidelines about Lp(a)<br><br>[GPs expressed confusion due to dissonance between international guidelines]	FG2 S1: "Honestly, I don't know. […] the Stanford people said that in America they don't care [about Lp(a)], Europe they care a bit more. […] what can a GP do?"
	Dissonance between local and international guidelines for hyperlipidemia<br><br>[GPs expressed confusion due to dissonance between local and international guidelines]	FG1 P8: "[…] and in fact, the ten-year coronary risk is kind of very lenient, quite a few patients I wanted to give — turns out the MOH tells me not to give statin. So, in fact, it's running opposite from what the Lp(a)'s group, which is to be a bit more stringent, maybe?"
Sub-category: Constraints are compounded in Polyclinic		
Difficulties with Lp(a) testing and management in Polyclinic setting<br><br>[Barriers faced by GPs are even more challenging in Polyclinic setting]	NA	FG1 P5: "I think for the polyclinic uh which I locum occasionally, their time is a big thing that make it very difficult. Their management is […] even less ideal."

APPENDIX

CATEGORY: Strategies for GP to Initiate Lp(a) Testing		
Sub-category: Education and training		
Code<br>[Description]	Sub-code (if any)<br>[Description]	Representative quotes from participants (quotes may appear in more than one code or sub-code if relevant)
Address knowledge gap<br><br>[GPs wanted evidence-based information about Lp(a)]	Questions about Lp(a)<br><br>[GPs had specific questions about Lp(a) that they would like information on]	Summary of questions from GPs about Lp(a) from the two focus groups:<br>• What is Lp(a)?<br>• How does it help in cardiovascular risk stratification?<br>• How would knowing that a patient has elevated Lp(a) change subsequent clinical management?<br>• What strategies and treatments are available now to address the increased cardiovascular risk of patients with elevated Lp(a)?<br>• Would the benefit of more aggressive treatment with statins outweigh the increased risk of developing diabetes?
	Targeted education events<br><br>[GPs were willing to learn about Lp(a) via education events such as CME and awareness campaigns]	FG1 P9: "We never even heard of Lp(a). So, unless we attend those CME talks that talk about Lp(a). Now we understand better […] there is some education for the public as well as for the general GPs, the older GPs, especially who are not aware of the importance."<br><br>FG2 S3: "I think it's good […] that's why I'm here [CME Forum] because I'm interested to know what else can we do, to help patient and myself as well."
Debunk misperceptions<br><br>[Misperceptions that need to be debunked/addressed via education and training]	Lp(a) testing confers theoretical advantage only<br><br>[GPs perceived that Lp(a) testing provides them with a theoretical advantage only; no effective treatment currently]	FG1 P4: "Because, you know, you can't do anything about it; the risk is there [elevated Lp(a)] but exercise won't change anything unless you have medication. But you don't have medication."

Provide patient engagement tools<br><br>[Provide tools GPs can use with their patients; for example, conversation scripts, visual aids/diagrams, booklets, pamphlets]	NA	Example of a conversation script — FG1 P9: […] just once in your life [Lp(a) test]. That's it. We will not repeat ever again, we'd know your risk factor already. […] So, maybe that will change their perspective about the money." (FG1 P9)<br><br>Example of patient-facing education materials — FG1 P7:"This [Lp(a) booklet provided to GPs at this CME Forum] is good enough. But also actually help us to show patient um that we are looking into this [Lp(a)], but I think at this moment of time, where we do recommend it — as I say, it is not for everybody — we should actually tell patient now there's no treatment for it [to lower Lp(a) levels]. It is telling us this [risk stratification] and that's […] most likely genetic."
Sub-category: Guidelines from Ministry of Health		
Guidelines for Lp(a) testing and management should be suitable for Singapore<br><br>[GPs wanted MOH guidelines that are suitable for the Singapore population, preferably based on local data/evidence]	NA	FG1 P8: "[…] we need more Singapore experience on this; because these criteria are all international. […] quite a few patients I wanted to give—turns out MOH tells me not to give statin. […] So, I think we need something of *[sic]* authority […] MOH to come out with better criteria, because these may be all international criteria."
Guidelines on cascade testing for elevated Lp(a)<br><br>[GPs wanted MOH guidelines on cascade testing for elevated Lp(a)]	NA	FG1 P4: "just follow, like the breast cancer guidelines, you know."
Sub-category: Subsidized Lp(a) test		
MOH guideline-enabled billing mechanisms	Access to subsidized Lp(a) test<br><br>[GPs would be able to bill Medisave or CHAS; essentially Lp(a) test subsidized or sponsored by the government]	FG1 P7: "So, it has to be from upstairs [MOH] and subsidized […]."

	Avoid out-of-pocket costs for patients<br><br>[Billing to Medisave or CHAS would avoid out-of-pocket costs for patients]	FG1 P7: "[…] because Medisave and FlexiMedisave, they don't see their money, they are quite happy. As long as they don't pay [out-of-pocket]."

THEME 4. WHAT PREVENTS & ENABLES PATIENTS TO ACCEPT Lp(a) TESTING

**CATEGORY: Patient Barriers for Accepting Lp(a) Testing**		
Sub-category: Knowledge gap		
Code<br>[Description]	Sub-code (if any)<br>[Description]	Representative quotes from participants (quotes may appear in more than one code or sub-code if relevant)
Lack of awareness<br><br>[General public and patients are not aware of Lp(a) and its role in cardiovascular risk assessment]	NA	FG1 P9: "I think there's an awareness issue. Because the public may not know what's the importance of Lp(a), and they think it is just extra cost, you know. After all, you've already done the fasting lipid."
Sub-category: Avoidance		
Patients don't want to know<br><br>[Some patients are afraid and prefer not to know]	NA	FG1 P3: "[…] we also need to consider individual choice […] people may be afraid […] there are people who tell me 'I really don't want to know'"
Avoid emotional distress<br><br>[Patients may resist Lp(a) test to avoid emotional distress]	NA	FG1 P3: "[…] someone [patient] actually said, 'after you do the testing for me, if it is abnormal, I have to start worry[ing] not just myself, [but for] my family, my children, and you know, everybody [then] needs to get tested.' […] there are implications because it's a genetic thing as well."
Sub-category: Trustworthiness of GP		
Patients don't question tests ordered in hospital<br><br>[Patients tend not to question the tests that are ordered in a hospital setting]	Patients trust public hospitals<br><br>[Patients tend to trust public hospitals]	FG1 P6: "[…] they trust the hospital, because after all it's under the government."

	"Decisions are made for you"<br><br>[In a hospital setting, patients tend to accept clinical decisions that are made for them]	FG1 P3: "[…] my patients go to the hospital ward, they get a lot [of] testing done, you know. [They] don't say anything, because the hospital doctors make decisions for you, the specialist makes decisions for you."
Patients question tests ordered by GPs when out-of-pocket costs are incurred<br><br>[Patients tend to question tests ordered by GPs, especially if out-of-pocket costs are incurred]	Justification needed<br><br>[GPs needed to provide justification that the Lp(a) test is necessary]	FG1 P3: "Whereas for us, it would take a lot of time to explain, it takes a lot of effort to explain; and it's not so easy."
	Not over-testing for profit-making<br><br>[GPs need to provide assurance that they are not over-testing to make a profit]	FG1 P6: "So, sometimes I just turn to the page [Family Medicine newsletter]; I'll underline and I'll show them [the data/evidence]. And then, they recognize that because it's from recognized institution, they are a little bit more open to it [Lp(a) test]."<br><br>FG1 P1: "[…] some patients will be upset […] testing something [Lp(a)] which, you know, […] there'll be some question […] or some profit […] from ordering a test that you cannot do [anything about]."
Sub-category: Affordability		
Cost of Lp(a) test<br><br>[Current cost of Lp(a) test prohibitive for some patients]	NA	FG1 P5: "I have not ordered the test […] those who are on CHAS, even if they are on a blue CHAS, and even if they have multiple illnesses, they can only claim up of $95 per visit [maximum claim]. If this test itself cost $55, it is very, very hard to convince them to do that."<br><br>FG1 P9: "[…] it may not be $55, maybe it's only $25, you know. Then, it's more acceptable to the masses."

APPENDIX

CATEGORY: Strategies to Promote Patient Acceptance of Lp(a) Testing		
Sub-category: Awareness and education		
Code<br>[Description]	Sub-code (if any)<br>[Description]	Representative quotes from participants (quotes may appear in more than one code or sub-code if relevant)
Address knowledge gap<br><br>[Address knowledge gaps of the general public and patients about hyperlipidemia and Lp(a)]	Education about hyperlipidemia<br><br>[Address knowledge gaps about hyperlipidemia and familial hyperlipidemia]	FG2 S8: "I always tell my patients there are two sources of cholesterol. Not just from your food, your liver makes it too. So, if you have familial hyperlipidemia, it's just a thermostat set higher; so that they'll make more [cholesterol]. […] so no matter how clean your diet is […] it's gonna be at that level."
	Education about Lp(a)<br><br>[Address knowledge gaps about Lp(a) and its utility in cardiovascular risk assessments]	FG1 P9: "[…] some education for the public […]"<br><br>FG1 P6: "I think actually, it's useful [Lp(a) test] because then the patient know, oh my risk if actually higher."
Authoritative education materials<br><br>[Education materials for the general public and patients should be from relevant government agencies or recognized institutions]	NA	FG1 P7: "[…] printed, with Health Promotion Board's logo; those are usually more convincing. […] If they [Health Promotion Board] take a lead, if they put up the article on their website, […] a pamphlet […] or booklet."<br><br>FG1 P5: "[…] Health Promotion Board should come up with brochures on this thing [Lp(a) testing] now."
Mass dissemination of education materials<br><br>[Dissemination of education materials via mass media and social media]	NA	FG1 P5: "[…] the media is very important […] MOH definitely recognizes that."
Sub-category: Subsidized Lp(a) test		
MOH guideline-enabled billing mechanisms<br><br>[Having MOH guidelines for Lp(a) testing and management would enable GPs to access billing	Access to subsidized Lp(a) test	FG1 P7: "So, it has to be from upstairs [MOH] and subsidized […]."

mechanisms resulting in either subsidized or no out-of-pocket costs for patients]	[GPs would be able to bill Medisave or CHAS; essentially Lp(a) test subsidized or sponsored by the government]	
	Avoid out-of-pocket costs for patients<br><br>[Billing to Medisave or CHAS would avoid out-of-pocket costs for patients]	FG1 P7: "[…] because Medisave and FlexiMedisave, they don't see their money, they are quite happy. As long as they don't pay [out-of-pocket]."
Sub-category: Financial incentives for preventive health		
Financial incentives<br><br>[Financial incentives in the form of reduced national health insurance premiums for achieving health goals]	NA	"Your cholesterol good, your diabetes good, your blood pressure good […] body fat good, 10% off [for each achievement]. […] age-appropriate physical fitness […] that is total 50%. You get 50% off Medishield Life." (FG2 S1)
